# The contribution of homestead pond fish culture to household food security and dietary diversity in central coast of a developing country

**DOI:** 10.1016/j.heliyon.2024.e28598

**Published:** 2024-03-28

**Authors:** M. Belal Hossain, F.H. Pingki, M. Sultana, N.M. Salim, M.M. Islam, A.F.M. Arifur Rahman, Bilal Ahamad Paray, Takaomi Arai

**Affiliations:** a Department of Fisheries and Marine Science, Noakhali Science and Technology University, Noakhali, 3814, Bangladesh; b School of Engineering and Built Environment, Griffith University, Brisbane, QLD, 4111, Australia; c Department of Food Technology and Nutrition Science, Noakhali Science and Technology University, Noakhali, 3814, Bangladesh; d Nutrition unit, Bangladesh Agricultural Research Council, Farmgate, Dhaka, 1200, Bangladesh; e Department of Zoology, College of Science, King Saud University, P.O. Box 2455, Riyadh, 11451, Saudi Arabia; f Environmental and Life Sciences Programme, Faculty of Science, Universiti Brunei Darussalam, Jalan Tungku Link, Gadong, BE1410, Brunei Darussalam

## Abstract

Fish farming in homestead ponds help alleviate poverty, provide animal source food, micronutrients, and indirect income and various jobs in developing nations. This study investigated the impact of homestead pond fish farming on dietary diversity (HDDS and MDD-W), food security (HFIAS and ELCSA), income, and women's engagement. A total of 185 households were selected randomly for data collection through well-structured questionnaire interviews in the central coast of Bangladesh. HDDS revealed significant dietary diversity (73.3%) among beneficiary farmers, surpassing controls and nearly doubling that of non-aquaculture farmers (41.1%). Additionally, this study found that 86.7% and 74.3% of women in beneficiary and homestead pond farmers exhibited high dietary diversity (MDD-W ≥ 5), whereas 48.6% of women in non-aquaculture farmers' households had low dietary diversity (MDD-W ≥ 5). Based on both ELCSA and HFIAS, higher prevalence of food security was observed among the beneficiary farmers that was about 60% and 63.3%, respectively compared with the control farmers. Most non-aquaculture farmers (62.9%) indicated their family consumed fish for one week before the research. More than half of the homestead pond culture (55.7%) and more than 90% of the beneficiary farmers, aquaculture farmers and non-aquaculture farmers had gross income (<$ 500). Pertaining to women's participation in homestead pond was positively correlated to productivity while male dominated tasks was negatively correlated with productivity. The results offer insights into how homestead pond fish farming can enhance food security by supplying direct animal protein, addressing protein and micronutrient deficiencies, and boosting income. The study emphasizes the urgent necessity for training and promoting homestead pond culture, increasing female participation, and advocating comprehensive support from governmental organizations (GOs) and non-governmental organizations (NGOs) to optimize production, improve micronutrient adequacy, and guarantee household food security. **Keywords**: Fish farming, food security, dietary diversity, women's participation.

## 1 Introduction

Food insecurity became a prime public health concern for not only the developing but also the developed ones [1,2]. The global challenge of hunger is escalating, impacting an estimated 821 million people worldwide, while two billion people face moderate or severe food insecurity [3]. In this context, Bangladesh holds the 90th position, with 27.1% experiencing a serious hunger situation. Furthermore, the increasing prevalence of micronutrient deficiencies poses a significant challenge to achieving the zero-hunger target in the Sustainable Development Goals by 2030. This challenge is exacerbated by insufficient intake of micronutrients, unbalanced diets, and low bioavailability [[4], [5], [6]]. Likewise, the extent and severity of both macro and micronutrient deficiencies significantly impact the long-term economic and human development of a nation [[6], [7], [8]]. Therefore, ensuring sufficient intake of both macro and micronutrients is essential in the fight against malnutrition.

Fishes are not only a good source of protein but also rich in several micronutrients such as vitamin A, vitamin B12, vitamin D, calcium, iodine, selenium and zinc [5,9]. About 60% people from developing countries depend upon fish for over 30% of their protein supplies [6,[10], [11], [12]]. Pond culture serves as the cornerstone of aquaculture in Bangladesh, constituting over 80% of the total documented production and encompassing more than 55% of the cultured area during the 2014–15 period [13]. Because of rapid expansion of aquaculture, polyculture system on the homestead pond can improve the consumption of micronutrients, household income and may create more employment opportunities thereby enhancing household food security [12,[14], [15], [16], [17]].

The close proximity of households offers a significant opportunity for women to actively engage and contribute to pond-based fish farming. This stands in contrast to other forms of aquaculture and capture fisheries, where women are frequently excluded due to cultural and social barriers, as well as the distant locations of these activities from their households [18,19]. Women have the potentiality to make up half of the workforce and play an eminent role in fisheries and aquaculture across the world through their presence in all steps of fish production activities such as pond preparation, weeding, stocking, fertilization, harvesting, processing etc. Therefore, women can contribute to the maintenance of households as well as rural communities through generating wealth in coastal regions [14,20].

Previous studies consistently underscore the crucial role played by homestead pond culture in addressing food security challenges by augmenting household dietary diversity and serving as a valuable source of animal protein [8,[21], [22], [23]]. The literature also accentuates the potential for income generation and livelihood improvement, particularly within low-income households engaged in pond aquaculture [5,12,17]. Furthermore, ecological benefits (such as wastewater recycling and nutrient utilization) associated with homestead pond culture, have been thoroughly investigated [5,6]. Nonetheless, dietary diversity, gender dynamics, and sustainability concerns emerge as challenges in the literature, emphasizing the necessity for a comprehensive and inclusive approach to promote and optimize the advantages of homestead pond culture in Bangladesh.

Previous research has investigated factors influencing household dietary diversity (HDDS) [19,21,23,24], minimum dietary diversity for women (MDD-W) [26], the Escala Latinoamericana y Caribeña de Seguridad Alimentaria (ELCSA) [27,28], Household Food Insecurity Access Scale (HFIAS) [29,30], and the involvement of women in pond farming [28,31] across various countries. However, these investigations did not analyze dietary intake on a household scale, dietary diversity of women, and additional constraints of measuring the household food insecurity access scale. These constraints encompass the assumption of local independence and limitations in data collection, such as household income and fish consumption. Hence, this study aims to evaluate the contribution of homestead pond fish culture in enhancing household dietary diversity and food security, while also examining women's participation in fish culture from the central coast of Bangladesh. A number of key concepts were theoretically covered when examining dietary diversity and household food security among small-scale fish farmers in coastal Bangladesh. Firstly, the livelihoods framework took into account the many livelihood strategies used by households engaged in fish farming, including fishing, agriculture, and non-farm pursuits. It also looked at how access to resources like human, financial, physical, natural, and social capital affects the results of food security. Secondly, the food systems approach looked at the food cycle, from production to consumption, taking into account how cultural preferences, market accessibility, and local food environments affect dietary variety and nutritional results. Thirdly, the impact of gender dynamics on household food security was examined, taking into account women's involvement and resource accessibility. Through the integration of these theoretical concepts, the study can offer a comprehensive understanding of the dietary diversity and household food security among small-scale fish farmers in coastal areas of Bangladesh. This understanding can then be used to inform policies and interventions targeted at enhancing food security and nutrition outcomes in these vulnerable communities.

## 2 Materials and methods

### 2.1 Study site and subjects

A cross-sectional study was conducted in six upazilas (Noakhali Sadar, Subarnachar, Hatiya, Ramgati, Kobirhat, Companiganj, Daganbhuiyan) within the greater Noakhali district, Bangladesh. A total of 185 households, predominantly comprising low-income homestead pond farmers, were randomly chosen through a multi-stage cluster sampling method from January to March 2021 in the central coast of Bangladesh. Multi-stage cluster sampling is a method used in survey research to efficiently collect data from diverse groups within a larger population. It involved a systematic process of selecting representative clusters, such as geographic regions or villages where fish farming was prevalent. Within each chosen cluster, a subset of fish farmers was randomly sampled, considering factors like diverse farming practices, income levels, and participation in specific programs. This method allowed to efficiently collect data that reflects the diversity of fish farming livelihoods within the broader population. Among these households, 30 were designated as beneficiary farmers who received support, including fish feed, fish seed, fertilizer, lime, technical assistance, and management guidance to engage in small-scale fish culture. Among the remaining 155 farmers, 70 households were engaged in aquaculture production, 70 households were non-aquaculture producers, and 15 households were involved in the aquaculture value chain.

### 2.2 Data collection

A draft questionnaire was developed based on the existing literature. The draft questionnaire was checked by the experts in the specific area of concern. The modified questionnaire was pre-tested among a subset of the study population who were not included in the main study. The final questionnaire was improved, rearranged and modified according to the information in the field testing (Supplementary Appendix- A). The final questionnaire included income, dietary diversity, household food insecurity scale measurement and women participation in homestead ponds of the respondents. To investigate women's participation, producers were asked about who was responsible for eight fish production tasks and who made decisions about adopting and expanding fish culture. In case of beneficiary and homestead pond farmers, only net income was included by subtracting the cost of fish feed, seed, fingerlings and labour from gross income. But monthly income data was collected by asking their income source for both non-aquaculture producers and aquaculture value chain workers. Ethical clearance was taken from the Research Cell, Noakhali Science and Technology University (Ref. No. 09/2019/RCNSTU-87).

### 2.3 Dietary diversity and food security

A popular method for evaluating the diversity and quality of foods consumed within a home over a certain length of time is the Households Dietary Diversity Score (HDDS). It offers a quick and easy way to assess nutritional sufficiency and eating habits inside the home. The diversity of food categories ingested by women of reproductive age over the course of a 24-h period is evaluated using the Minimum Dietary Diversity for Women (MDD-W) metric [[25], [32], [33]]. It functions as a gauge of this demographic group's nutritional sufficiency and quality of food. For assessing food security among farmers, the Escala Latinoamericana y Caribeña de Seguridad Alimentaria (ELCSA) offers a method that is particular to the specific area and culture. Using a thorough methodology, ELCSA evaluates a range of aspects of food security and provides in-depth information on household food access, availability, and consumption [34]. Because of its uniform approach, it can be compared across different agricultural communities, which helps policymakers create targeted measures that effectively fight food poverty [12,28,35]. In contrast, Household Food Insecurity Access Scale (HFIAS) is a widely used comprehensive and consistent method for evaluating food security among farmers, making comparison and analysis simple. Its culturally sensitive questions guarantee accuracy and relevance, and its quantitative results help policymakers create effective, focused responses. Furthermore, HFIAS permits longitudinal monitoring, which enables researchers to monitor changes in the state of food security over time and assess the effectiveness of interventions [35].

In this study, HDDS consisting of 12 food groups was used to determine the household dietary diversity and MDD-W constituting 10 food groups was used to determine the dietary diversity of women (Swindale and Bilinsky, 2006; FAO, 2016). In addition, the overall food security was assessed through a well-structured questionnaire consisting of 15 questionnaires of which first 8 were used to investigate for adults in the preceding three months according to ELCSA [28,32,35] which is adapted for Latin America from the Household Food Insecurity Access Scale [35]. Furthermore, the household food security status was also assessed using HFIAS (FANTA v.3) [35]. Each query is a yes/no format. Each "yes" response is worth one point. The total number of points represents the severity of food insecurity in the household. In the ELCSA assessment, scores of 0 indicate food security, while scores of 1, 2, and 3 correspond to mild, moderate, and severe levels of food insecurity, respectively. Similarly, in the degree of food insecurity assessment (HFIAS), a score of 1 represents food security, while scores of 2, 3, and 4 signify mild, moderate, and severe levels of food insecurity access, respectively.

### 2.4 Statistical analysis

All data have undergone a thorough examination, cleaning, and meticulous summarization. Statistical analyses were conducted using the Statistical Package for Social Science (SPSS) version 25. Analysis of Variance (ANOVA) was employed to ascertain variations in mean dietary diversity scores and household food insecurity. Pearson correlation (r) was utilized to elucidate the association between income and dietary diversity scores, as well as household food insecurity scores (HFIAS and ELCSA) within each group. Additionally, Spearman rank correlation (rs) was performed to assess the correlation between net income derived from homestead pond fish culture and the involvement of both male and female participants in fish culture activities.

## 3 Results and discussion

### 3.1 Household dietary diversity

HDDS was computed for all farmers, including both beneficiaries and controls, while MDDW was excluded for aquaculture value chain workers due to the absence of women in that category. The analysis revealed substantial dietary diversity (73.3%) among beneficiary homestead pond farmers, surpassing control farmers and nearly doubling the dietary diversity of non-aquaculture control farmers (41.1%) (Table 1). In a study by Ahmed et al. (2019), 35% of tea workers in two Sylhet tea gardens were found to have low dietary diversity, indicating that small-scale aquaculture might play a more significant role in nutrition for those workers compared to the findings in this study. Homestead pond farmers (70%) consumed six or more food groups, followed by non-aquaculture farmers (41.4%) and aquaculture value chain workers (66.7%) (Table 1).

**Table 1:** Prevalence of dietary diversity and food security among the households.

	Beneficiary farmers n (%)	Aquaculture producer n (%)	Non-aquaculture farmers n (%)	Value chain workers n (%)
HDDS				
Low (1–3 food groups)	3 (10)	6 (8.6)	16 (22.9)	0
Medium (4 & 5 food groups)	5 (16.7)	15 (21.4)	25 (35.7)	5 (33.3)
High (>5 food groups)	22 (73.3)	49 (70)	29 (41.4)	10 (66.7)
**MDD-W**				
Low (<5 food groups)	4 (13.3)	18 (25.7)	33 (47.1)	
High (≥5 food groups)	26 (86.7)	52 (74.3)	37 (52.9)	
**ELCSA**				
Food secure	18 (60)	19 (27.1)	6 (8.6)	2 (13.3)
Mild food insecurity	6 (20)	27 (38.6)	32 (45.7)	8 (53.3)
Moderate food insecurity	5 (16.7)	15 (21.4)	25 (35.7)	5 (33.4)
Severe food insecure	1 (3.3)	9 (12.9)	7 (10)	0
**HFIAS**				
Food secure	19 (63.3)	22 (31.4)	5 (7.2)	2 (13.3)
Mildly food insecure access	7 (23.3)	26 (37.1)	25 (35.7)	7 (46.7)
Moderately food insecure access	4 (13.3)	16 (22.9)	26 (37.1)	6 (40)
Severely food insecure access	0	6 (8.6)	14 (20)	0

Note: n = number, % = percentage, HDDS = Household Dietary Diversity Score, MDD-W = Minimum Dietary Diversity for Women, ELCSA = Escala Latinoamericana y Caribena de Seguridad Alimentaria, HFIAS = Household Food Insecurity Access Scale.

In the Amatole and Nyandeni districts of South Africa, Taruvinga et al. [36] discovered that 29.3%, 35.9%, and 34.8% of households of pond fish farmers exhibited low, medium, and high dietary diversity scores, respectively. This disparity from the findings of our study among non-aquaculture farmers can be attributed to differences in geographical locations, as well as variations in academic environments, among other factors.

There was a significant (p < 0.05) difference in household dietary diversity between beneficiary and control farmer households. Moreover, in comparison to other livelihoods, the impact of domestic fish farming on improving dietary diversity was relatively modest. However, it's important to note that a high score for dietary diversity does not necessarily imply desirability or nutritional benefits, as highlighted by Swindale and Bilinsky [32]. According to our findings, non-aquaculture farmers had a lower HDDS (mean of 5.54 out of 12) than beneficiary and control farmers, such as homestead pond farmers (n = 6.57) and aquaculture value chain workers (n = 6.53). While the score does not directly measure whether or not a household eats a good or unhealthy diet, it is thought to be indicative of the household's ability to afford both.

Table 2 displays the proportion of households from each respondent group that consumed items within each HDDS food category. The result of the present study showed 90% beneficiary farmers, 95.7% homestead pond farmers and 95.7% non-aquaculture farmers consumed cereals respectively which indicates that their diet was pre-eminently based on cereals. The results of the current study align with prior research by Mbwana et al. [28] and Irwin et al. [37], highlighting the prevalence of cereals in diets. Fish consumption was notably higher (93.3%) among beneficiary farmers compared to control farmers, with homestead pond farmers (61.4%) and value chain workers (66.7%) exhibiting greater fish consumption than non-aquaculture farmers. This trend may be attributed to the on-farm accessibility of fish, potentially making it a more affordable dietary option for homestead pond farmers and value chain workers. The similar findings also reported by the previous study of Irwin et al. [28] (n = 106) in Bolivia among aquaculture producer, non-aquaculture farmers and value chain workers. Results from the Kruskal-Wallis tests, ‘White tubers and roots, ‘fish and other sea food’ as well as “legumes, nuts and seeds were the only food group categories where there was a significant difference in mean consumption between groups.

**Table 2:** Proportion of Households from each respondent group that consumed an item in each Household Dietary Diversity (HDDS) food category and measurement of difference between groups.

Respondent group						
**HDDS food Category**	**Beneficiary farmers n (%)**	**homestead pond farmers** **N (%)**	**Non-aquaculture producers n (%)**	**Aquaculture value chain workers n (%)**	**Kruskal – Wallis H**	***p*-Value**
Cereals	27 (90)	67 (95.7)	67 (95.7)	15 (100)	2.564	0.464
White tubers and roots	2 (6.7)	42 (60)	31 (44.3)	4 (26.7)	25.99	0.000
Vegetables	19 (96.7)	47 (67.1)	50 (71.4)	11 (73.3)	9.93	0.019
Fruit	13 (43.3)	31 (44.3)	23 (32.9)	6 (40)	2.14	0.54
Meat	15 (50)	41 (58.6)	24 (34.3)	3 (20)	12.49	0.006
Eggs	15 (50)	26 (37.1)	26 (37.1)	8 (53.3)	2.79	0.426
Fish and other seafood	28 (93.3)	43 (61.4)	29 (41.4)	10 (66.7)	24.03	0.000
Legumes, nuts and seeds	5 (16.7)	19 (27.1)	6 (8.6)	10 (66.7)	26.55	0.000
Milk and milk products	23 (76.7)	31 (44.3)	26 (37.1)	4 (26.7)	15.83	0.001
Oils and fats	25 (83.3)	64 (91.4)	65 (92.9)	10(100)	4.041	0.257
Sweets	14 (46.7)	27 (38.6)	25 (35.7)	7 (46.7)	1.41	0.70
Spices, condiments and beverages	15 (50)	22 (31.4)	16 (22.9)	5 (53.3)	7.18	0.066

Note: n = number, % = percentage, HDDS = Household Dietary Diversity Score.

#### 3.1.1 Minimum dietary diversity for women (MDD-W)

It is important to record the consumption of women at the individual level because women usually consume less variety of foods than men [38]. Hence, to estimate the micronutrient adequacy of in the diet of women within households, the MDD-W indicators were evaluated (Table 1). The findings reveal that among beneficiary farmers and homestead pond farmers, 86.7% and 74.3% of women, respectively, achieved an MDD-W score equal to or above 5. In contrast, among non-aquaculture farmers, 48.6% of women had an MDD-W score below 5. This findings of the beneficiary and homestead pond fish farmers was consistent with the study of Hanley et al. [39] where 50.5% women had minimum dietary diversity score below 5 among the non-pregnant women of reproductive age (WRA) in Cambodia, Ethiopia and Zambia. Significant differences between groups were observed in the mean minimum dietary diversity score for women (*p* < 0.05).

The present study revealed no significant differences on the consumption of ‘grains, white roots and tubers, and plantains and dairy foods between the beneficiary and control farmers (Table 3). However, significant differences (p < 0.05) were observed among the groups in terms of their pulses (beans, peas and lentils); nuts and seeds; meat, poultry and fish; vitamin A-rich fruits and vegetables; other vegetables and other fruits consumption. Consumption of eggs (41.4%), meat, poultry and fish (37.1%) and vitamin A-rich fruits and vegetables (45.7%) were significantly less consumed (p < 0.05) among the women in non-aquaculture farmer's household compared to the beneficiary farmers, homestead pond farmers and aquaculture value chain workers (Table 3), may be due to not having their own farm (Table 3). The food group consumption patterns among women in households of homestead pond fish farmers align with the findings of a prior study by Islam [40]. However, the current study observed that 37% of respondents consumed eggs, 35% consumed meat, and 34.76% consumed vegetables rich in vitamin A.

**Table 3:** Proportion of women in the households consumed foods from Medium Dietary Diversity for Women (MDD-W) food categories.

Respondent group					
MDD-W food categories	Beneficiary farmers n (%)	Homestead pond fish farmers n (%)	Non-aquaculture producers n (%)	Kruskal – Wallis H	*p* Value
Grains, White roots and tubers, and Plantains	30 (100)	70 (100)	70 (100)	0.000	1.000
Pulses (beans, peas and lentils)	29 (96.7)	32 (45.7)	13 (18.6)	74.78	0.000
Nuts and seeds	17 (56.7)	15 (21.4)	18 (25.7)	15.32	0.000
Dairy	16 (53.3)	51 (72.9)	46 (65.7)	3.60	0.165
Meat, poultry and Fish	29 (96.7)	38 (54.3)	26 (37.1)	41.59	0.000
Eggs	20 (66.7)	43 (61.4)	29 (41.4)	11.59	0.003
Dark green leafy vegetables	19 (63.3)	33 (47.1)	50 (71.4)	2.55	0.280
Other vitamin A-rich fruits and vegetables	10 (33.3)	57 (81.4%)	32 (45.7)	27.48	0.000
Other vegetables	21 (70)	30 (42.9)	12 (17.1)	38.45	0.000
Other fruits	21 (70)	13 (18.6)	24 (34.3)	21.62	0.000

**Note:** n = number, % = percentage, MDD-W = Minimum Dietary Diversity for Women.

#### 3.1.2 Household food security

Based on ELCSA and HFIAS assessment, majority of households in all groups faced some degree of food insecurity (Table 1). Assessment of ELCSA implies a likely trend of highest food security among the beneficiary and control farmers except non-aquaculture farmers. Beneficiary farmers exhibited a higher prevalence of food security (60%), contrasting with the dominant occurrence of food insecurity (91.4%) among non-aquaculture farmers. These findings align with the results reported in the study by Ref. [28]. Significant difference in food security (p < 0.05) was observed among the three groups.

Based on the findings of HFIAS, it was found that the household of the beneficiary farmers had the highest food security (63.3%) based food insecurity was highest among the non-aquaculture farmers (92.8%) (Table 1). Our findings are in line with the study McDonald et al. (2015) for non-aquaculture farmers with dominant food insecure households. Nonetheless, a comparable study [41] that also deployed the HFIAS documented a slightly greater incidence of household food security (18%) in the four rural districts (n = 900) of Cambodia in 2015 than the value chain workers (13.3%) in our study. Surprisingly, based on both ELCSA and HFIAS, no severe food insecurity was found among the aquaculture value chain workers may be due to having direct source of fish and other vegetables consumption from on-farm access like beneficiary farmers. Food security causes vary by country and culture. Food insecurity is caused by long-term poverty and a lack of productive resources, livestock ownership, monthly income, family size, structure household education, size, livestock ownership, market access, and remittances. Low income also contributes to family food insecurity since families cannot afford enough food [42].

### 3.2 Respondent fish consumption

This study revealed the dominance of fish consumption among the beneficiary and control farmers except non-aquaculture farmers during the previous 24 h of reference period (Table 4). When the non-aquaculture farmers were asked about their consumption of fish during the previous week of the study date, majority of them (62.9%) answered that their family ate fish during the previous week (Table 4). They mentioned that their source of fish acquisition was either directly from a fisher/farmer or from the market in comparable proportions. Most of the non-aquaculture farmers (47.1%) indicated that market was their prime source of fish acquisition whereas 81.4% consumed fish at home, a finding consistent with research on the contribution of small-scale aquaculture on food security in Bolivia [28].

**Table 4:** Details of fish consumption of non-aquaculture farmers (n = 70).

Household fish consumption	n (%)
Households that reported eating fish regularly	2 (2.9)
Households that ate fish during the previous week	44 (62.9)
Households that ate fish during the previous week and regularly	17(24.3)
Households that didn't eat fish during the previous week or regularly	7 (10)
Number of times households ate fish the previous week	Mean 6.54; range 0–21
**Location of fish acquisition**	
Purchased directly from farmer or fisher	6 (8.6)
Purchased from a market	33 (47.1)
Obtained from a river	1 (1.4)
River and market	22 (31.4)
Fisher and market	8 (11.4)
**Location of fish consumption**	
Home	57 (81.4)
Restaurant	0
At a friend's home	2 (2.9)
Home and restaurant	11 (15.7)

Note: n = number, % = percentage.

### 3.3 Indirect effect of homestead pond fish farming on food security through income

#### 3.3.1 Respondent income

**Fig. 1:**
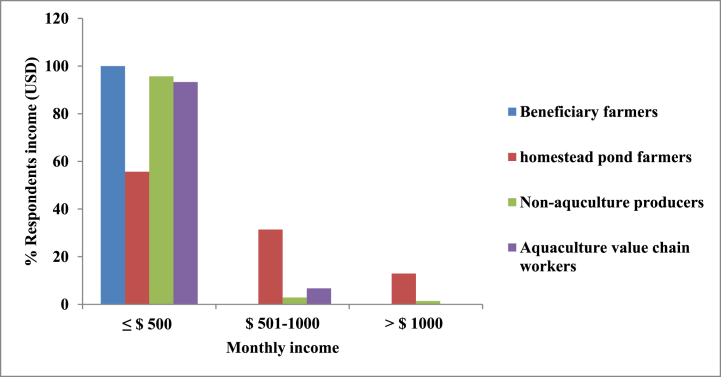
Distribution of the households according to monthly income.

The average net income of the beneficiary farmers during the last harvested production cycle was $134.60 ± 66.59 USD while for the homestead pond farmers net income from fish farming during the last harvested production cycle ranged from $48.81 USD to a profit of $1476.19 USD. The mean was $499.16 USD, the median was $445.24 USD, and the standard deviation was $369.84 USD. For non-aquaculture farmers and aquaculture value chain workers, monthly average gross income was observed about $191.85 ± 289.49 USD and $157.94 ± 129.41 USD, respectively. Fig. 1 shows the percent distribution of the households according to their monthly income. It was evident that the monthly income of all the beneficiary farmers was ≤ $500 USD. Besides, about 58%, 31% and 12% of the homestead pond farmers had average monthly income ≤ $500 USD, $501–1000 USD and > $1000 USD, respectively. This aligns with the national average of approximately 1.5 tons of fish per hectare per season. The variability in income is attributed to the considerable differences in the size of aquaculture operations, measured in square meters of pond area. Larger operational areas tend to yield higher fish production, resulting in increased income. Belton and Azad [43] indicated that homestead ponds serve as a notable indirect income source, with reported production levels ranging from 1.5 to 5.5 tons of fish per hectare per season. The net income derived exclusively from homestead pond farming by both beneficiary and homestead pond farmers was contrasted with the gross income of non-aquaculture farmers and aquaculture value chain workers, encompassing all their livelihood activities [8,21]. For homestead pond farmers, the net income solely from fish culture closely approximated the gross income of non-aquaculture farmers, who received income from various household livelihood activities. This suggests that, in addition to other income sources, homestead pond farmers, including both beneficiaries and non-beneficiaries, achieved a net income comparable to the gross income of non-aquaculture households, indicating a relatively higher household income from aquaculture compared to non-aquaculture households [8]. Regarding the beneficiary farmers, net income from homestead pond aquaculture was strongly, positively and significantly correlated (*r =* 0.786*, p =* 0.000) with m^2^ of stocked pond. Productivity (net income/m^2^) of stocked pond ranged from $0.23 USD to $0.84 USD with a mean of $0.5146 USD and a median of $0.5148 USD (data not shown). Net income per m^2^ of stocked pond was strongly, positively and significantly correlated to the cost of inputs (labour, fingerlings and feed) per m^2^ *(r =* 0.837*, p =* 0.000). In context of homestead pond farmers, the correlation between net income and m^2^ of stocked pond was found to be moderately, positively and significantly correlated; *r* = 0.453, *p* = 0.00. Productivity (net income/m^2^) of stocked pond ranged from 0.11 USD to 6.65 USD with a mean of 1.49 USD and a median of 0.76 USD. Net income per m^2^ of stocked pond was moderately, positively and significantly correlated to the cost of inputs (labour, fingerlings and feed) per m^2^ (*r* = 0.554*, p* = 0.00).

To evaluate the significance of aquaculture, the aquaculture value chain workers were asked about their previous profession and how they would compare it with their present profession to evaluate the significance of aquaculture among them (Table 5). The majority of value chain workers expressed satisfaction with their current profession in fish culture, considering it to be an improvement over their previous occupation. This sentiment aligns with the findings of Irwin et al. [28], who observed that aquaculture value chain workers generally perceived their employment as slightly better than jobs in other food value chains.

**Table 5:** Frequency of aquaculture value chain workers’ responses to questions comparing previous employment to current aquaculture value chain employment.

Questions	A lot less n (%)	A little less n (%)	The same n (%)	A little more n (%)	A lot more n (%)
Do you consider the money you make at this job to be a lot less, a little less, the same, a little more, or a lot more than the money you made at your previous job?	0	0	1(6.7)	8 (53.6)	6 (40)
Do you consider the overall quality of this job to be a lot worse, a little worse, the same, a little better, or a lot better than that of your previous job?	0	1(6.7)	1(6.7)	2 (13.3)	11 (73.3)
	Very poor	Poor	Not so poor	Well off	Rich
Before you began this job, did you consider yourself to be very poor, poor, not so poor, well off, or rich?	3 (20)	5 (33.3)	6 (40)	7 (6.7)	0
Today, do you consider yourself to be very poor, poor, not so poor, well off, or rich?	1 (6.7)	1 (6.7)	2 (13.3)	10 (66.7)	1 (6.7)

Note: n = number, % = percentage.

#### 3.3.2 Association of income, food security and dietary diversity

The association between monthly income and household dietary diversity and food security were assessed. For the beneficiary farmers, significant positive correlation was observed between their monthly income and HDDS, MDD-W and HFIAS. Besides, for the homestead pond farmers, monthly income from the pond culture was insignificantly correlated to HDDS, MDD-W and HFIAS. In case of the non-aquaculture farmers, gross income was insignificantly correlated with MDD-W and HFIAS, while net income from aquaculture and gross income from non-aquaculture was also found to be insignificantly correlated with ELCSA. The above findings revealed that monthly income of homestead pond fish culture had modest effect on household food security and this finding was in line with the previous study conducted by Irwin et al. [28] among the small-scale aquaculture producers in Bolivia.

### 3.4 Women's participation in homestead pond farming and the effect on food security

#### 3.4.1 Women's participation in homestead pond farming

To know the women's participation in homestead pond fish culture of central coast, they were asked who in their households had made the decision to start farming fish. Participants were also asked who was responsible for the tasks linked with fish production. Male involvement in homestead pond fish culture activities was more prevalent than female participation, although both genders engaged simultaneously in activities for both beneficiary and homestead pond fish farmers (Table 6). In case of the beneficiary farmers, the mean number of tasks headed by only male was about 3.53 ± 4.07 whereas the average for female was 2.53, with a standard deviation of 2.88. The mean number of tasks headed by both male and female was 2.93, with a standard deviation of 2.69. Regarding to homestead pond farmers, the mean number of tasks responsible solely by male was 4.11, with a standard deviation of 2.98, whereas the average for female was 1.47, with a standard deviation of 1.89. The mean number of tasks headed by both male and female was 3.41, with a standard deviation of 2.69. Among nine production tasks to culture in homestead pond, 46.7% of feeding tasks were executed by female as well as 53.5% of feeding tasks were executed by both male and female. Moreover, 96.7% and 93.3% male contributed in pond construction and fingerling procurement, respectively. But male (100%) and female (100%) did marketing and processing, respectively. Castine et al. [14] demonstrated that 55% and 72% of female contributed in feeding and pond construction, miscellaneous tasks, respectively concerning to the homestead pond which is more agreed with the present findings in feeding but inconsistent with pond construction executed by female in beneficiary farmers. In the perspective of human, female participation in fish production tasks is viewed as great variation in different area.

**Table 6:** Frequency distribution of the aquaculture production activities headed by male, female or both.

Activity	Person responsible (Gender)	Beneficiary farmers (n)	homestead pond farmers (n)	Beneficiary farmers (%)	homestead pond farmers (%)
Pond construction n = 70	Male	29	43	96.7	61.4
	Female		6	3.3	8.6
	Both	1	21		30
Fingerling procurement n = 70	Male	28	56	93.3	80
	Female		3		4.3
	Both	2	11	6.7	15.7
Feeding n = 70	Male		9		12.9
	Female	14	12	46.7	17.1
	Both	16	49	53.3	70
Pond maintenance n = 70	Male	13	37	43.3	52.9
	Female	6	14	20	20
	Both	11	19	36.7	27.1
Monitoring of fish growth n = 70	Male	1	5	3.3	7.1
	Female	10	8	33.4	11.4
	Both	19	57	63.3	81.4
Harvesting n = 70	Male		43		61.4
	Female	11	8	36.7	11.4
	Both	19	19	63.3	27.1
Processing n = 70	Male		11		15.7
	Female	30	49	100	70
	Both		10		14.3
Marketing n = 70	Male	30	70	100	100
	Female	0	0	0	0
	Both	0	0	0	0
Record keeping n = 70	Male	5	14	16.7	20
	Female	5	3	16.7	4.3
	Both	20	53	66.6	75.7

Note: n = number, % = percentage.

Concerning homestead pond farmers, exclusive female participation was noted in processing (70%), deviating from the findings of Irwin et al. [28]. Additionally, a higher proportion (11.4%) of harvesting tasks was undertaken by females, surpassing the observations of Castine et al. [14] due to variations in the study area. The current results highlight the significant role of women in homestead pond culture activities, such as feeding and processing, contributing to household income. This female involvement not only increased productivity and profitability but also enhanced overall household security compared to non-aquaculture farmers. Similar positive contributions of women in small-scale aquaculture, boosting production in Bangladesh, have been evidenced, supporting the connection between women's participation in aquaculture and improved food security [44]. Thus, the findings suggest the potential for women to engage in this income-generating activity, thereby supporting various sectors such as healthcare, food purchasing, and childrens' schooling. Moreover, more or less contribution of female was found with male in aquaculture production tasks. In the absence of men, feeding, processing and pond maintenance is not time and labour intensive because of the location of the pond nearest to the home, though it dovetails greatly with many of the other household obligations that women have such as household and childcare management with other social constraints to engage in aquaculture activities. The practice of keeping financial records solely by women was not prevalent and was considered a positive approach in the study area, but it was nearly evenly distributed between men and women. The activities including fingerling procurement, harvesting, marketing and monitoring in which females were least often involved. In such case, this task is labour intensive, disliked by female and hence traditionally carried out by male.

#### 3.4.2 Women's participation and food security

We investigated the potential correlation between the involvement of men, women, or joint efforts in up to nine fish culture activities (as detailed in Table 6) and the household's food security and dietary diversity score (Table 7) for both beneficiary and homestead pond farmers. However, no statistically significant correlation (p > 0.05) was observed, consistent with the findings of Irwin et al. [28].

**Table 7:** Spearman correlations between gendered aquaculture production responsibilities, household dietary diversity score, Medium dietary diversity for women, degree of food security (**ELCSA)**.

	Activities are performed solely by a man		Activities are performed solely by a woman		Activities where both man and woman are involved	
	Beneficiary farmers	Homestead pond farmers	Beneficiary farmers	Homestead pond farmers	Beneficiary farmers	Homestead pond farmers
**HDDS**	*rs* = 0.270 (*P* = 0.483)	*rs* = 0.275 (*P* = 0.473)	*rs* = - 0.365 (*P* = 0.334)	*rs* = −0.017 (*P* = 0.965)	*rs* = 0.017 (*P* = 0.965)	*rs* = −0.131 (*P* = 0.736)
**MDD-W**	*rs* = 0.299 (*P* = 0.434)	*rs* = 0.018 (*P* = 0.964)	*rs* = - 0.419 (*P* = 0.262)	*rs* = 0.075 (*P* = 0.848)	*rs* = 0.343 (*P* = 0.366)	*rs* = −0.386 (*P* = 0.309)
**ELCSA**	*rs* = 0.017 (*P* = 0.965)	*rs* = 0.240 (*P* = 0.533)	*rs* = 0.026 (*P* = 0.947)	*rs* = 0.215 (*P* = 0.579)	*rs* = - 0.227 (*P* = 0.556)	*rs* = −0.053 (*P* = 0.891)

Note: HDDS = Household Dietary Diversity Score, MDD-W = Minimum Dietary Diversity for Women, ELCSA = Escala Latinoamericana y Caribena de Seguridad Alimentaria.

#### 3.4.3 Women's participation and income

Further we examined if there was a correlation between the number of activities that was performed by solely women and men with household net income from homestead pond fish cultures, the size of fish culture operations as measured by m^2^ of pond surface, and the productivity of fish culture operations as measured by productivity (income/m^2^) of pond area (Table 8).

**Table 8:** Association (Spearman correlations) between the number of tasks man and woman do alone and monthly income, size of operation, and productivity from fish culture.

	Number of tasks performed solely by man		Number of tasks performed solely by woman	
	Beneficiary farmers	Homestead pond farmers	Beneficiary farmers	Homestead pond farmers
**Net income**	*rs* = 0.034 (*p* = 0.931)	*rs* = 0.167 (*p* = 0.667)	*rs* = 0.051 (*p* = 0.896)	*rs* = −0.084 (*p* = 0.830)
**Size of operation**	*rs* = 0.026 (*p* = 0.947)	*Rs* = 0.257 (*p* = 0.504)	*rs* = - 0.017 (*p* = 0.965)	*rs* = −0.051 (*p* = 0.897)
**Productivity**	*rs* = - 0.153 (*p* = 0.695)	*rs* = 0.092 (*p* = 0.814)	*rs* = 0.254 (*p* = 0.509)	*rs* = 0.084 (*p* = 0.830)

In case of sole task performed by woman in beneficiary households, weak, insignificant and positive correlation was observed with productivity (p > 0.05), but the correlation was very close to being moderate and significant (Table 8). This indicates that households where women participate in the management of aquaculture operations with men are likely to achieve greater success compared to those solely managed by women or men alone. This finding aligns with a study conducted by Irwin et al. [28] among small-scale aquaculture producers in Bolivia.

### 3.5 Limitations and recommendations for future work

The outcomes of this study are site-specific, applicable primarily to the central coast of Bangladesh, thereby constraining the broader generalizability of the results to other geographical locations with distinct socio-economic and environmental dynamics. The extensive categorization of income levels (<$500) may oversimplify the economic diversity within this cohort, potentially complicating various factors that could influence the association between income and observed outcomes. To gain a more inclusive understanding of trends and causality, future investigations should consider employing longitudinal studies to capture temporal variations in dietary diversity, food security, income, and women's engagement. Furthermore, expanding the geographical scope of the study to encompass diverse regions would augment the external validity of the findings, offering a more inclusive perspective on the implications of homestead pond fish farming.

## 4 Conclusion

The objective of this study was to assess how homestead pond fish culture contributes to enhancing household food security and promoting women's participation in fish culture in the central coast region of Bangladesh. The study reveals that based on Household Dietary Diversity Score (HDDS) and Minimum Dietary Diversity for Women (MDD-W), beneficiary farmers and control farmers (excluding non-aquaculture farmers) exhibit the highest dietary diversity, including a greater consumption of fish within the past 24 h. Notably, non-aquaculture farmers demonstrate higher levels of food insecurity compared to beneficiary farmers, homestead pond farmers, and aquaculture value chain workers. The diversified on-farm access of aquaculture value chain workers contributes to preventing acute food insecurity. Importantly, the findings highlight that household pond fish farming plays a crucial role in enhancing dietary diversity and fulfilling animal protein requirements. While income showed no significant correlation with dietary diversity or food security across groups except gross income, the study emphasizes that income is a pivotal factor influencing both high dietary diversity and household food security. The study underscores the need for comprehensive support, including homestead pond aquaculture training, gender-sensitive approaches, and extension services provided by governmental and non-governmental organizations, to optimize production, enhance micronutrient adequacy, and ensure sustained household food security. Additionally, the study sheds light on the surprising negative connection between male-dominated pond culture and production, emphasizing the indirect financial and nutritional benefits derived from the active participation of women in domestic pond fish cultivation activities. Despite the accessibility of household ponds, the study indicates limitations on the productivity of women, reinforcing the importance of targeted interventions and gender-sensitive approaches to maximize production and ensure holistic food security. These findings can guide targeted interventions and policies to enhance sustainable aquaculture practices, ensuring improved nutrition and livelihoods within vulnerable communities.

## Funding

This study was supported by Food Based Project, PIU, NATP-2, 10.13039/100010719BARC (Grant ID-011), Bangladesh. This study was partially supported by the 10.13039/100009100Universiti Brunei Darussalam under the Faculty/Institute/Centre Research Grant (No. UBD/RSCH/1.4/FICBF(b)/2023/057). In addition, this study was supported by Researchers Supporting Project Number (RSP2024R144), 10.13039/501100002383King Saud University, Riyadh, Saudi Arabia.

## Ethical clearance

This study was approved by the Ethical Committee, Research Cell, of Noakhali Science and Technology University (Ref. No. 09/2019/RCNSTU-87).

## Data availability statement

Data will be available upon reasonable request.

## CRediT authorship contribution statement

**M. Belal Hossain:** Writing – review & editing, Writing – original draft, Supervision, Funding acquisition, Conceptualization. **F.H. Pingki:** Writing – original draft, Formal analysis, Data curation. **M. Sultana:** Investigation, Formal analysis, Data curation. **N.M. Salim:** Investigation, Formal analysis, Data curation. **M.M. Islam:** Resources, Investigation, Funding acquisition. **A.F.M. Arifur Rahman:** Methodology, Funding acquisition, Formal analysis. **Bilal Ahamad Paray:** Funding acquisition, Formal analysis, Data curation. **Takaomi Arai:** Writing – review & editing, Funding acquisition.

## Declaration of competing interest

The authors declare no competing interest.
