# Supplementary material for: The contribution of homestead pond fish culture to household food security and dietary diversity in central coast of a developing country
Source: Heliyon. 2024 Mar 28;10(7):e28598. doi: 10.1016/j.heliyon.2024.e28598 (PMC10990951; doi:10.1016/j.heliyon.2024.e28598)
Supplement: Multimedia component 1 [file mmc1.docx]

**Supplimentary file (Appendex -A)**

**The contribution of homestead pond fish culture to household food security and dietary diversity in central coast of a developing country**

M. Belal. Hossain ^1,2,^*, F. H. Pingki ^1^, M. Sultana^3^, N. M. Salim^1^, M. M. Islam^4^, A. F. M. Arifur Rahman^1^, Bilal Ahamad Paray^5^, Takaomi Arai^6^

^1^ Department of Fisheries and Marine Science, Noakhali Science and Technology University, Noakhali 3814, Bangladesh.

^2^ School of Engineering and Built Environment, Griffith University, Brisbane, QLD 4111, Australia.

^3^ Department of Food Technology and Nutrition Science, Noakhali Science and Technology University, Noakhali 3814, Bangladesh.

^4^ Nutrition unit, Bangladesh Agricultural Research Council, Farmgate, Dhaka-1200, Bangladesh.

^5^ Department of Zoology, College of Science, King Saud University, P.O. Box 2455,
Riyadh 11451, Saudi Arabia.

^6^ Environmental and Life Sciences Programme, Faculty of Science, Universiti Brunei Darussalam, Jalan Tungku Link, Gadong BE1410, Brunei Darussalam.

* Correspondence: [mbhnstu@gmail.com](mailto:mbhnstu@gmail.com) (M.B.H)

# QUESTIONNAIRE

Respondent no: Date:

Time:

# Background Information:

- 1. Name:

# Address:

a. Union: b. Village: c. Upazila: d. District:

# Fish farming status

- 1. Pond size (m^2^)

| Small (< 200) |  |
| --- | --- |
| Medium (200–325) |  |
| Large (>325) |  |

- 1. Pond depth (m)

| Low (1–2) |  |
| --- | --- |
| High (>2) |  |

- 1. Pond ownership:

|  | single ownership |  | Multiple ownership |
| --- | --- | --- | --- |

- 1. Pond type

|  | seasonal |  | perennial |
| --- | --- | --- | --- |

- 1. Culture type

|  | monoculture |  | polyculture |
| --- | --- | --- | --- |

- 1. Harvesting frequency

|  | Total harvest |  | Partial harvest |
| --- | --- | --- | --- |

- 1. Purpose of fish production

| Purpose of fish production | No. of category |
| --- | --- |
| Personal |  |

| Sell |  |
| --- | --- |
| Both |  |

# Demographic profile of homestead pond fish farmers in the central coastal area.

1. **Human capital**
   1. Religion

|  | Islam |  | Hindu |  | Buddish |  | Others |
| --- | --- | --- | --- | --- | --- | --- | --- |

- 1. Household size

| Household size | No. of category |
| --- | --- |
| Small (<4) |  |
| Medium (5–6) |  |
| Large (>7) |  |

- 1. Family type

|  | Joint |  | Nuclear |
| --- | --- | --- | --- |

- 1. Education

| Education level | No. of category |
| --- | --- |
| Illiterate |  |
| Primary |  |
| Secondary |  |
| Higher secondary |  |
| Bachelor |  |

# Financial capital

- 1. Sources of credit:

| Credit sources | No. of category |
| --- | --- |
| Self |  |
| Bank |  |

| Relatives |  |
| --- | --- |
| NGOs |  |

- 1. Occupation:

| Occupation | Primary occupation | Secondary occupation |
| --- | --- | --- |
| Fish culture |  |  |
| Agriculture |  |  |
| Business |  |  |
| Day labour |  |  |
| Poultry raising |  |  |
| Others |  |  |

- 1. Annual average income:

| Income range (USD) | Number |
| --- | --- |
| ≥500 |  |
| 500.01–1000.00 |  |
| 1000.01–1500.00 |  |
| 1500.01–2000.00 |  |
| >2000.00 |  |

# Physical capital

- 1. Housing condition

| Housing condition | No. of category |
| --- | --- |
| Kaccha |  |
| Tin-shed |  |
| Half-cemented |  |
| Cemented |  |

- 1. Land area (m^2^)

| Land area (m^2^ ) | No. of category |
| --- | --- |
| Small (<850) |  |
| Medium (850-2025) |  |
| Large (>2025) |  |

- 1. Health facilities

| Health facilities | No. of category |
| --- | --- |
| Village |  |
| Upazila |  |
| MBBS |  |
| Kobiraj |  |
| No facility |  |

- 1. Drinking water sources

| Drinking water sources | No. of category |
| --- | --- |
| Own |  |
| Neighbour |  |
| Pond and tube well |  |
| River |  |

- 1. Sanitation facilities:

| Sanitation facilities | No. of category |
| --- | --- |
| Kaccha |  |
| Semi-pacca |  |
| Pacca |  |

- 1. Electric facilities:

|  | Yes |  | No |
| --- | --- | --- | --- |

- 1. Length of time involved in fish farming (years)

| Length of time | No. of category |
| --- | --- |
| Self study |  |
| Friends |  |
| Relatives |  |
| NGOs |  |

- 1. Training and experience on fish farming

| Training and experience on fish farming |  |
| --- | --- |
| Self |  |
| Friends |  |
| Upazila |  |
| NGOs |  |

3.. Livelihood outcome

|  | Positive outcome |  | Negative outcome |
| --- | --- | --- | --- |

1. Constraints faced by the pond fish farmers

………………………………………………………………………………………………

……………………………………………………………………………………………… Signature of the interviewer

………………………………………………….
